# The Efficacy of Multivitamin, Vitamin A, Vitamin B, Vitamin C, and Vitamin D Supplements in the Prevention and Management of COVID-19 and Long-COVID: An Updated Systematic Review and Meta-Analysis of Randomized Clinical Trials

**DOI:** 10.3390/nu16091345

**Published:** 2024-04-29

**Authors:** Alessandra Sinopoli, Antonio Sciurti, Claudia Isonne, Maria Mercedes Santoro, Valentina Baccolini

**Affiliations:** 1 Local Health Authority Roma 1, Department of Prevention, 00193 Rome, Italy; 2 Department of Public Health and Infectious Diseases, Sapienza University of Rome, 00185 Rome, Italy; 3 Department of Experimental Medicine, University of Rome “Tor Vergata”, 00133 Rome, Italy

**Keywords:** vitamin, COVID-19, systematic review, randomized clinical trials

## Abstract

This review aims to evaluate the efficacy of any vitamin administration(s) in preventing and managing COVID-19 and/or long-COVID. Databases were searched up to May 2023 to identify randomized clinical trials comparing data on the effects of vitamin supplementation(s) versus placebo or standard of care on the two conditions of interest. Inverse-variance random-effects meta-analyses were conducted to estimate pooled risk ratios (RRs) and 95% confidence intervals (CIs) for all-cause mortality between supplemented and non-supplemented individuals. Overall, 37 articles were included: two regarded COVID-19 and long-COVID prevention and 35 records the COVID-19 management. The effects of vitamin D in preventing COVID-19 and long-COVID were contrasting. Similarly, no conclusion could be drawn on the efficacy of multivitamins, vitamin A, and vitamin B in COVID-19 management. A few positive findings were reported in some vitamin C trials but results were inconsistent in most outcomes, excluding all-cause mortality (RR = 0.84; 95% CI: 0.72–0.97). Vitamin D results were mixed in most aspects, including mortality, in which benefits were observed in regular administrations only (RR = 0.67; 95% CI: 0.49–0.91). Despite some benefits, results were mostly contradictory. Variety in recruitment and treatment protocols might explain this heterogeneity. Better-designed studies are needed to clarify these vitamins’ potential effects against SARS-CoV-2.

## 1. Introduction

Viral infections are a significant global health concern [1]. They can lead to a wide range of illnesses, from the common cold to more severe and lethal diseases like influenza but also acquired immunodeficiency syndrome or COVID-19 [1,2]. Public health practices [3], including handwashing, social distancing, and other non-pharmacological treatments, are a cornerstone in reducing the spread of viral infections and minimizing their impact on individuals and communities [4]. Combined with vaccinations, they have mitigated the effects of the COVID-19 pandemic, during which searching for any effective supplemental treatment or preventive measure rekindled the interest in the role of micronutrients [5,6].

It is well-known that vitamins play a crucial role in infections [7] as they are essential for the proper functioning of the immune system. Clinical studies show that vitamin D deficiency is associated with a higher risk of respiratory infections, including COVID-19 [8]; some authors have reported the positive effect of vitamin A supplementation on the risk of severe diseases via an immunomodulatory and anti-inflammatory effect, replicable for COVID-19 [9,10], while a review has concluded that some reduction in duration and severity of common cold symptoms can be observed in regular supplementation trials [11].

As research progressed during the pandemic, data on the effects of vitamin administration in contributing to managing COVID-19 have accumulated [12,13,14,15] but evidence is still inconclusive [12,14] and their role in the prevention of the infection has not been systematically investigated to date. Additionally, some studies have explored the potential role of vitamins in managing the symptoms of long-COVID and post-acute sequelae of SARS-CoV-2 infection but with no clear conclusion [16]. Therefore, this systematic review and meta-analysis aimed to update the synthesis of evidence on the role of any vitamin supplementation, in any form or administration route, in the prevention and management of COVID-19 and/or long-COVID. The results could help clarify the clinical effects of these dietary supplements against SARS-CoV-2.

## 2. Materials and Methods

This systematic review was conducted according to the Cochrane Handbook for Systematic Reviews and the Preferred Reporting Items for Systematic Reviews and Meta-Analyses statement [17,18]. The review protocol was registered at PROSPERO, identifier CRD42022362055. Since this study did not involve primary data collection, the protocol was not submitted for institutional review board approval and did not require informed consent.

### 2.1. Inclusion and Exclusion Criteria

Eligible articles were randomized clinical trials (RCTs) conducted in any country, published in English or Italian, that compared data on the direct effects between (i) vitamin administration in any form, dosage, and route of administration and (ii) placebo or standard of care, in relation to the prevention and/or management of COVID-19 and/or long-COVID in people of any age. The following essential vitamins were considered eligible for inclusion [19], alone or in combination: vitamin A, vitamin C, vitamin D, vitamin E, vitamin K, vitamin B1 (thiamine), vitamin B2 (riboflavin), vitamin B3 (niacin), vitamin B5 (pantothenic acid), vitamin B6 (pyridoxine), vitamin B7 (biotin), vitamin B9 (folic acid), and vitamin B12 (cyanocobalamin). Long-COVID was defined as the continuation or development of new symptoms three months after the initial SARS-CoV-2 infection, with these symptoms lasting for at least two months and no other explanation [20].

When a vitamin(s) was administered with other substances, the study was considered eligible only when the vitamin(s) effect could be isolated. Non-randomized trials, observational studies, studies using in vitro techniques, and studies conducted on animals, focusing only on the vitamin’s capacity to stimulate the participants’ immune response without a confirmed SARS-CoV-2 infection or investigating any indirect effect (i.e., any effect in non-supplemented individuals, such as the outcomes of vitamin supplementation in children born from women receiving the nutrient) were excluded.

### 2.2. Search Strategy

Two reviewers independently searched PubMed, Scopus, and Web of Science, from database inception to 26 May 2023. MedRxiv.org and bioRxiv.org were interrogated as pre-print databases using the “medrxivr” R package [21]. The following key terms were used: COVID-19; long-COVID; SARS-CoV-2; vitamin*; and provitamin*. The string was adapted to fit the search criteria of each database (Table S1). No filter was applied in the search strategy. Duplicate articles due to database overlap were removed and the titles and abstracts of the collected records were screened. Studies that clearly did not meet the inclusion criteria were excluded. Full texts of potentially relevant articles were retrieved and independently examined by two researchers. Disagreements were resolved through discussion and reasons for exclusion were recorded. The reference lists of retrieved articles were also manually searched to identify other potentially relevant studies.

### 2.3. Data Collection, Quality Assessment, and Data Synthesis

For each eligible study, two reviewers independently extracted the following information: general characteristics of the trial (i.e., first author, year of publication, and country); characteristics of the intervention (i.e., target population, sample size, vitamin administered, vitamin status at baseline, vitamin dose, vitamin route, vitamin frequency of administration, and follow-up time); area of evaluation (i.e., prevention or management of COVID-19 and/or long-COVID); main findings; and side effects or adverse events. Effects were classified into four categories: (i) immunological, hematological, and laboratory outcomes; (ii) clinical outcomes; (iii) length of hospitalization; and (iv) all-cause mortality. Articles providing data on different outcomes but from people enrolled in the same trial were grouped.

Two independent authors performed the quality assessment of the articles included in the systematic review using the revised Cochrane Risk-of-Bias tool version 2 for randomized studies [22]. Any discrepancy was resolved by consensus.

Then, given the high heterogeneity of the intervention protocols applied and the outcomes investigated, for each vitamin/multivitamin complex, a narrative synthesis of the main results was performed, distinguishing the two areas of evaluation (i.e., prevention or management of COVID-19 and/or long-COVID). In addition, for vitamins C and D, separate inverse-variance random-effects meta-analyses were performed to estimate pooled risk ratios (RRs) and 95% confidence intervals (CIs) for all-cause mortality, comparing the cumulative incidence of deceased patients between the intervention and control group. The Cochrane I^2^ metric was used to quantify heterogeneity. It was considered statistically significant at *p*-value < 0.05 and substantial heterogeneity was defined as I^2^ > 50% [23]. In the main analysis, we stratified by time-interval (i.e., mortality ≤ 14 days vs. mortality > 14 days from enrollment) considering the longest follow-up available, while in sensitivity analyses, whenever possible, we stratified by hospitalization setting (i.e., intensive care unit (ICU)-hospitalized patients vs. hospitalized patients vs. non-hospitalized patients), vitamin status at baseline (i.e., 100% vitamin deficient patients vs. other), route of administration (i.e., oral vs. intravenous administration), and frequency of administration (i.e., single vs. multiple administrations). Lastly, the small-study effect, potentially caused by publication bias, was investigated by visual inspection through funnel plot asymmetry.

Analyses were performed using Review Manager (RevMan, version 5.4, The Cochrane Collaboration, Copenhagen, Denmark), R Statistical Software (version 4.2.3; R Core Team 2023, R Foundation for Statistical Computing, Vienna, Austria), and the package “meta” [24].

## 3. Results

**Figure 1:**
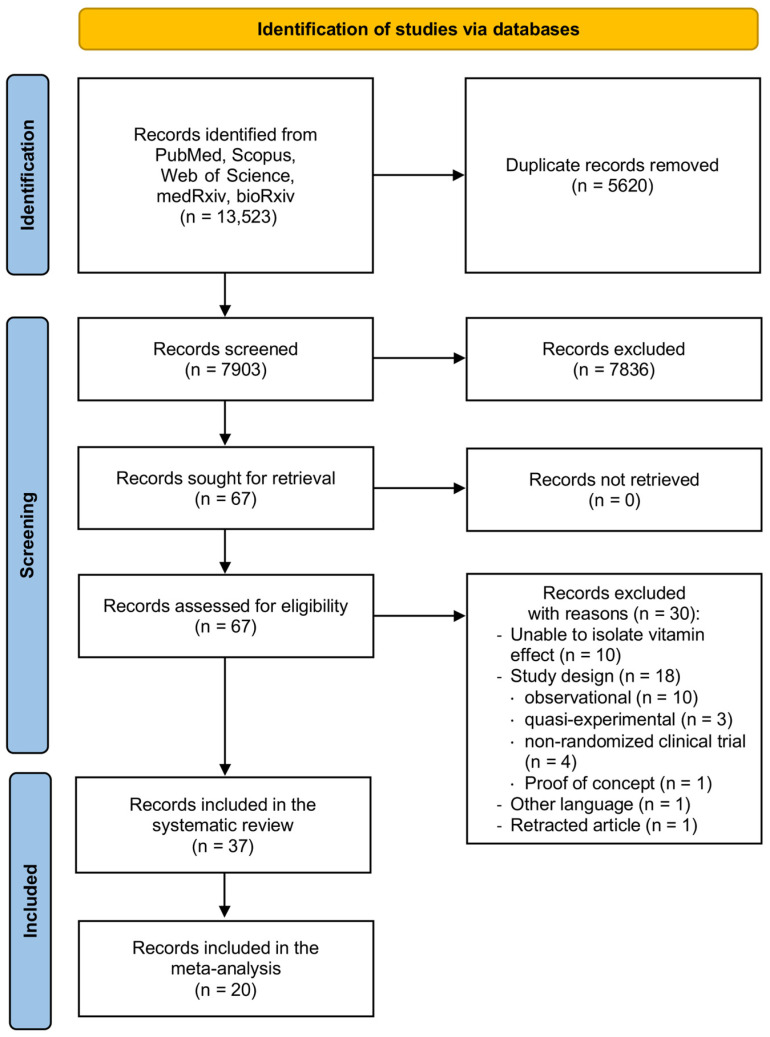
Flow diagram of the review process.

Overall, 13,523 records were identified by database search (Figure 1). After duplicate removal and screening by title and abstract, 67 articles were selected as eligible for full-text analysis, from which 30 were excluded with reasons, for a total of 37 articles ultimately included in the systematic review and 20 articles meta-analyzed.

### 3.1. Characteristics of the Included Studies in the Prevention of COVID-19 and/or Long-COVID

Two studies [25,26] published in 2022 and conducted in the United Kingdom [25] and Mexico [26], respectively, investigated vitamin D supplementation in the prevention of COVID-19, one of which extended the analysis to include long-COVID data [25] (Table 1). While one study [25] recruited a large number of non-hospitalized subjects (over 5000 people), the other RCT [26] enrolled a smaller sample of healthcare workers (less than 350 participants). In both cases [25,26], approximately three-quarters of the sample were females, while the proportion of individuals with vitamin D deficiency ranged from 67% in one trial [26] to 100% in the other RCT [25]. Intervention protocols included the oral supplementation of cholecalciferol/vitamin D3 with different dosages, from 800 international units (IU) [25] to 4000 IU [26], administered once daily for six months [25] or 30 days [26], with a follow-up of 6 months [25] and 45 days [26], respectively. The risk of bias was judged as high in both studies [25,26] (Table S2).

### 3.2. Characteristics of the Included Studies on the Management of COVID-19 by Vitamin Type

#### 3.2.1. Vitamin Co-Administration

Three studies investigated multivitamin supplementation in managing adult patients with COVID-19, two of which were conducted in Iran [9,27] and one in Mexico [28] (Table 2). The number of individuals enrolled was small in all trials (less than 100 patients), with the proportion of females ranging from 35% [28] to 49% [9]. The baseline levels of vitamins were measured in one case only [9]. Protocol interventions were heterogeneous: in one study [9], vitamin A, vitamin D, vitamin E, vitamin C, and vitamin B-complex were administrated intravenously with a varying dosage and frequency (from four times a day to only once in 7 days); in the second trial [27], vitamins C and E were administered orally once a day until discharge; in the last study [28], vitamins C and D were administered orally two times a day for 21 days. Follow-up ranged from 7 [9] to 40 days [28] or until hospital discharge [27]. The overall risk of bias was considered with some concerns [9], high [27] and low [28] (Table S2).

#### 3.2.2. Vitamin A

Two studies conducted in 2022 in Iran examined vitamin A supplementation in the management of COVID-19 in hospitalized [30] and non-hospitalized patients [29], respectively (Table 2). In both cases, the sample was small (less than 200 participants) with a female proportion of around 40%. Vitamin A levels were not tested at baseline but it was supplemented intramuscularly at a dosage of 50,000 IU in hospitalized patients [30] and orally at half of the dosage in outpatients [29]. In both studies, patients took vitamin A daily, with a similar duration of treatment (two weeks in hospitalized patients and 10 days in non-hospitalized patients). Participants were followed until hospital discharge [30] or for 10 days [29]. The overall risk of bias was low [30] and with some concerns [29] (Table S2).

#### 3.2.3. Vitamin B

Vitamin B supplementation in COVID-19 management was investigated in two studies conducted in 2022 in Iran [31] and in China [32] (Table 2). Participants were enrolled among hospitalized patients with COVID-19 [32] and in one case they were recruited from the ICU setting [31]. The population was small, ranging from 24 [32] to 85 [31] individuals, of whom about 50% were females. The levels of vitamin B at baseline were not assessed in any study and the route of administration was specified only in one case [31], in which vitamin B was supplemented intramuscularly. Vitamin form and dosage differed between the two studies as well as the duration of treatment and follow-up. Specifically, in one case, vitamin B complex was supplemented daily for two weeks with an additional two weeks of follow-up [31], while only nicotinamide was administered five times daily for a shorter time and follow-up (two days for both) [32]. The overall risk of bias was considered with some concerns [31] and high [32] (Table S2).

#### 3.2.4. Vitamin C

A total of 11 RCTs analyzed vitamin C supplementation in the COVID-19 management (Table 2). They were all conducted between 2020 and 2022 in Iran (n = 4) [35,38,39,41], the United States (n = 3) [33,34,42], India (n = 1) [36], Pakistan (n = 1) [37], Australia and Turkey (n = 1) [40], and China (n = 1) [43]. The sample size was relatively small, ranging from 54 [41] to 237 adults [40]. Participants were mainly recruited among hospitalized patients with moderate or severe COVID-19 (n = 6) [33,35,37,38,40,41], in three cases among ICU patients [36,39,43], and in the other two studies among non-hospitalized adults with mild or moderate COVID-19 [34,42]. The female proportion ranged from 22% [36] to 80% [34]. Vitamin C levels were not tested at baseline in any study but it was administered orally in three cases [34,39,42] and intravenously in the other eight studies [33,35,36,37,38,40,41,43]. The dosage was heterogeneous, from 0.5 g [36] to 12 gr [38], similar to the frequency of administration, from once daily for 5 days [33,34,39,42] to 4 times/day for at least 5 days [35,40,41]. Patients were followed until hospital discharge in seven studies [33,35,36,37,38,39,41] and for approximately one month in the other cases [34,40,42,43]. The risk of bias was judged as high in six studies [34,35,37,39,40,41], with some concerns in two trials [42,43], and low in the remaining three cases [33,36,38] (Table S2).

#### 3.2.5. Vitamin D

In total, 17 articles referring to 15 RTCs reported data on vitamin D supplementation in COVID-19 management (Table 2). They were conducted in Tunisia (n = 1 RCT) [44], the United States (n = 2 RCTs) [45,50], Croatia (n = 1 RCT) [46], Russia (n = 2 RCTs) [47,52], Belgium (n = 1 RCT) [49], Spain (n = 1 RCT) [51], Iran (n = 1 RCT) [53], Argentina (n = 1 RCT) [54], India (n = 1 RCT) [58], Mexico (n = 2 RCTs) [59,60], Brazil (n = 1 RCT, n = 3 papers) [55,56,57], and one RCT in four countries [48] (Table 2). The population enrolled was relatively small, ranging from 40 [58] to 240 individuals [55,56,57], with a varying proportion of females, from 37% [48] to 72% [46]. All but three trials [44,45,59] recruited hospitalized patients with mild, moderate, or severe COVID-19 and, among these, only one study considered the pediatric population [60]. Vitamin D at baseline was assessed in nine RCTs, with the proportion of vitamin D-deficient individuals ranging from 48% [55] to 100% [49,53,58,60]. Vitamin D was always administered orally in the form of cholecalciferol/vitamin D3 in 11 RCTs [44,46,47,48,49,52,54,55,58,59,60], calcifediol in 3 trials [45,51,53], and calcitriol in the remaining 1 RCT [50]. Dosage and frequency of administration were strongly heterogeneous: a single administration in four RCTs [44,48,54,55], two administrations in one trial [52], once daily for 7–14 days [46,50,59,60] or 60 days [53] in three cases, respectively, and in RCTs it consisted of de-escalation schedules [45,47,49,51,58]. Follow-up time ranged from 9 days [52] to one year [44,57] but about half of the studies followed participants until hospital discharge [46,47,48,50,51,54,55]. In seven papers [45,53,54,56,57,58,60], the risk of bias was judged as high, in the other seven studies [44,46,48,49,50,52,59] with some concerns, and in the remaining three cases [47,51,55] low (Table S2).

### 3.3. Main Findings of Vitamin Administration in the Prevention of COVID-19 and/or Long-COVID

One of the two articles [25] that analyzed the effects of administering vitamin D in COVID-19 and long-COVID prevention compared a high dose of supplementation, a low dose of supplementation, and no supplementation at all, while the other RCT [26] compared a high dose of supplementation to a placebo (Table 3). While vitamin D administration seemed to not influence the prevention of long-COVID symptoms at month six in the only study that analyzed it [25], data about the prevention of COVID-19 risk were contrasting, with one study [25] reporting a non-significant difference between treated and untreated in the prevention of SARS-CoV-2 infection, risk of hospitalization, and mortality at six months, whereas the Mexican RCT [26] found a significantly lower proportion of infections among vitamin D-supplemented individuals. Side effects did not differ between the intervention and control groups in both studies [25,26].

### 3.4. Main Findings of Vitamin Administration in the Management of COVID-19 by Vitamin Type

#### 3.4.1. Vitamin Co-Administration

In the three trials investigating multivitamin supplementation, the combination of vitamins was compared with no supplementation in two cases [9,28] or with the standard of care [27] (Table 4). Results were contrasting: a reduction in some inflammatory parameters in supplemented patients was registered in one study [9] but not in the other [27], whereas Leal-Martínez et al. [28] reported a significantly higher hydric balance and lower gastrointestinal distension among treated individuals but a non-significant difference in post-COVID syndrome, weight decrease, and gastrointestinal symptoms at day 40 from enrollment. Similarly, in the intervention group, a lower gravity score and length of hospitalization were found in the trial by Beigmohammadi et al. [9]; a lower mortality and higher saturation were registered by Leal-Martínez et al. [28], while Hakamifard et al. [27] did not report any difference between the two arms in clinical outcomes such as ICU-admission rate, temperature, and pulse rate, apart from a lower respiratory rate that was reported in patients who had received vitamins C and E. Side effects were not reported in the only study in which they were assessed [9].

#### 3.4.2. Vitamin A

The effects of vitamin A were evaluated by comparing its administration with the standard of care with [29] or without a placebo [30] (Table 4). No significant difference was found in the trial of Somi et al. between the two groups, neither in clinical outcomes such as ICU-admission rate, need for invasive or non-invasive mechanical ventilation, time for clinical response and treatment strategies, nor in mortality and in length of hospitalization. By contrast, a significant improvement in some clinical symptoms such as fever, body aches, weakness, and fatigue and also significant differences in immunological response were detected in the other trial [29]. Only one study [30] investigated side effects and found no events.

#### 3.4.3. Vitamin B

The effects of vitamin B were investigated by comparing the administration of vitamin B complex with nutritional complex without vitamin B in one trial [31] and vitamin B with the standard of care in the other one [32] (Table 4). No immunological difference was detected in both trials between the two arms, as well as no difference in hematological parameters or in organ function indexes [31,32]. Similarly, clinical outcomes such as oxygenation parameters, clinical aggravation, and mortality showed no difference between groups in the two studies [31,32]. The effects of vitamin B on the duration of hospitalization were not evaluated in the studies, in which the side effects were also not investigated [31,32].

#### 3.4.4. Vitamin C

The effects of vitamin C were mainly evaluated by comparing its administration with the standard of care, with [36,38,43] or without a placebo [33,35,37,41,42] (Table 4). No difference was observed in the four trials [38,39,41,43] investigating immunological response and other hematological and laboratory parameters, except for lower levels of Interleukin-6 detected after one week among patients treated with vitamin C in the study of Zhang et al. [43]. All studies assessed the clinical outcomes: of these, five trials [34,36,38,39,43] did not find any difference in severity scores, including SOFA (sequential organ failure assessment) or GCS (Glasgow Coma Scale), while five trials [33,35,36,37,43] did not report any difference in clinical improvement, oxygenation parameters, need for invasive or non-invasive mechanical ventilation, intubation, or admission to the ICU. By contrast, three authors [35,41,43] registered better respiratory parameters during the first day of hospitalization among supplemented individuals, even though the overall clinical improvement was not found to differ in two trials [42,43], was faster in one case [37], or was greater in another study [40].

As for length of hospitalization, four trials found no difference between the intervention and control group [33,38,41,43], one study [37] found it was shorter among treated individuals, whereas another study [35] found that it was longer, but with no difference after restriction to the ICU-admitted patient subgroup. Out of the six studies that analyzed side effects [33,35,39,40,42,43], only one found a higher proportion of events in the intervention group [42].

**Figure 2:**
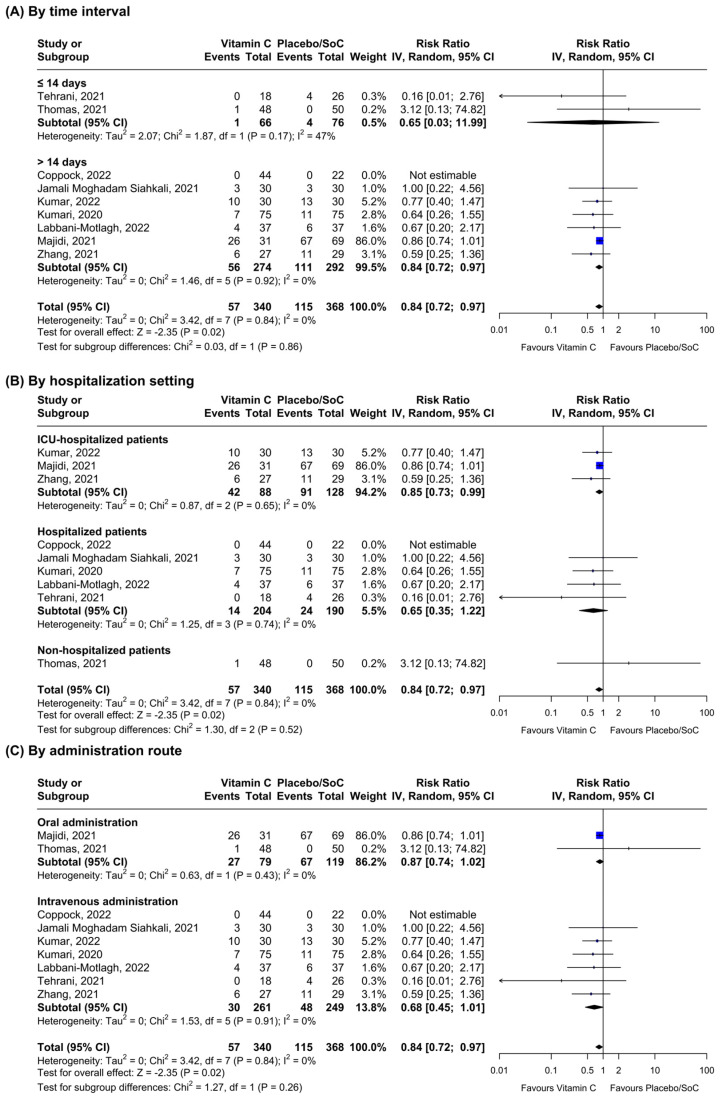
Stratified inverse-variance random-effects meta-analyses for all-cause mortality comparing patients receiving vitamin C vs. placebo or standard of care (SoC) [33,35,36,37,38,39,41,42,43].

Regarding all-cause mortality, nine RCTs provided data on the outcome and were included in the meta-analysis (Figure 2). Vitamin C supplementation seemed to reduce mortality in the overall analysis (n = 9, RR = 0.84; 95% CI: 0.72–0.97, I^2^ = 0.0%) and in the subgroup in which mortality was quantified after 14 days from enrollment (n = 7, RR = 0.84; 95% CI: 0.72–0.97, I^2^ = 0.0%). Sensitivity analyses by hospitalization setting showed significant results among ICU-hospitalized patients only (n = 3, RR = 0.85; 95% CI: 0.73–0.99, I^2^ = 0.0%), whereas vitamin C administration did not lead to reduced mortality in any group following stratification by administration route (oral supplementation: n = 2, RR = 0.87; 95% CI: 0.74–1.02, I^2^ = 0.0%, and intravenous supplementation: n = 6, RR = 0.68; 95% CI: 0.45–1.01, I^2^ = 0.0%, respectively). Heterogeneity between the subgroups was always non-significant (*p* > 0.05). Funnel plot analysis showed some evidence of asymmetry (Figure S1).

#### 3.4.5. Vitamin D

Vitamin D administration was compared to placebo in five trials [44,45,47,54,55], to standard of care in two studies [46,59], and no supplementation in two RCTs [48,52], whereas it was added to standard of care (with or without placebo) in the remaining six trials [49,50,51,53,58,60] (Table 5). Eight authors [45,46,48,51,55,57,58,59] did not report a difference between the intervention and control group in any of the immunological, hematological, and laboratory parameters investigated, whereas three trials [47,52,53] registered better outcomes during hospital stay but only in a few factors, including neutrophil cell count, neutrophil-to-lymphocyte ratio, and natural killer cell count. A significantly longer duration of viral RNA conversion was also reported in the supplemented group by Abroug et al. [44]. As for the clinical effects, severity scores such as SOFA were not found to differ [54], whereas some respiratory parameters were better among treated individuals in two cases [49,50] but not in another trial [52]. A need for intubation, respiratory support, and ICU admission rate were non significantly different between the two groups in most cases [46,47,48,49,50,52,54,55,56], even though two trials reported positive results favoring supplemented individuals [48,60]. Signs or symptoms of greater clinical improvement during hospital stay or at discharge were mentioned in two [49,59] out of the six RCTs that investigated such outcomes [44,45,46,48,49,59]. Furthermore, no difference was found in the duration of infection or the proportion of patients with re-infection at one year between the two groups [44,59]. Length of hospitalization was found to be higher among supplemented individuals in one trial [47], it was shorter in another trial [49], and did not differ in the other RCTs, considering both the overall patients [46,49,50,53,55,56], only those admitted to the ICU [46,49,53,54], or only those with vitamin D deficiency at baseline [52,55,56]. Side effects were not detected in five trials [46,47,49,50,53] or were not found to differ in the other four RCTs that were investigated [51,54,55,60].

**Figure 3:**
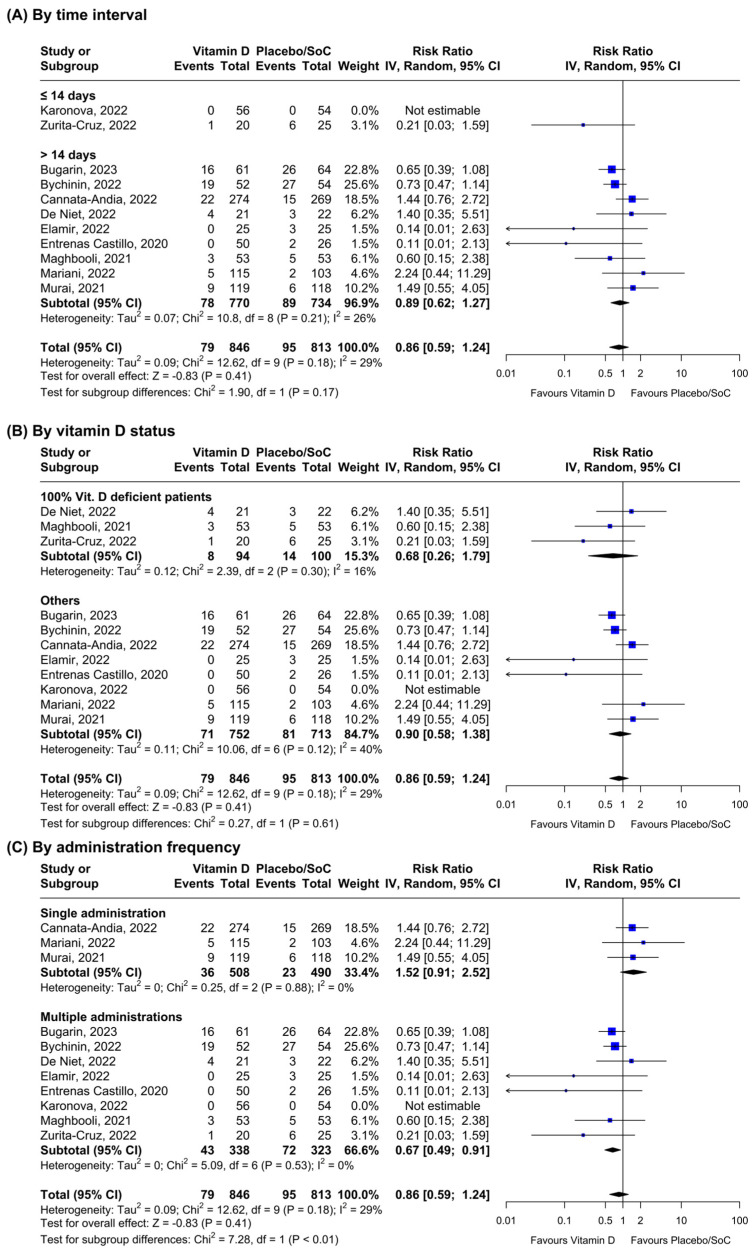
Stratified inverse-variance random-effects meta-analyses for all-cause mortality comparing patients receiving Vitamin D vs. placebo or standard of care (SoC) [46,47,48,49,50,51,52,53,54,55,60].

As for all-cause mortality, 11 RCTs provided data on vitamin D and all-cause mortality (Figure 3). The results did not show any significant reduction in the outcome incidence in the overall (n = 10, RR = 0.86; 95% CI: 0.59–1.24, I^2^ = 29%) and time-stratified analyses (mortality ≤ 14 days: n = 1, RR = 0.21; 95% CI: 0.03–1.59; and mortality > 14 days: n = 9, RR = 0.89; 95% CI: 0.62–1.27, I^2^ = 26%, respectively). At sensitivity analyses, vitamin D supplementation did not seem to reduce mortality according to the vitamin level at baseline (100% vitamin D deficient patients: n = 3, RR = 0.68; 95% CI: 0.26–1.69, I^2^ = 16%; and others: n = 7, RR = 0.90; 95% CI: 0.58–1.38, I^2^ = 40%, respectively). However, multiple administrations of vitamin D seemed to have a positive effect on the outcome (n = 7, RR = 0.67; 95% CI: 0.49–0.91, I^2^ = 0%) compared to the single administration subgroup (n = 3, RR = 1.52; 95% CI: 0.91–2.52, I^2^ = 0%). Heterogeneity between the subgroups was always non-significant apart from the frequency of administration stratification (*p* < 0.01). Funnel plot analysis revealed moderate asymmetry (Figure S2).

## 4. Discussion

Despite growing evidence [61,62] that proves the role of supplement compounds in supporting the immune system, the debate about the use of natural agents in the prevention and management of viral infections is still far from solved [63,64,65]. Indeed, although it is well-known that vitamins are critical to making the immune system work properly [66,67], the results coming from clinical trials on the role of these substances in COVID-19 disease are inconclusive [13,68]. It is not surprising that many reviews have already been published on the topic [13,15,69,70] but they mostly focused on one vitamin only [14,15,68,71] or a specific outcome in COVID-19 patients [13,69,70]. Alternatively, or in addition, they had different inclusion criteria [12,14,68], such as the investigation of vitamin administration in combination with other substances or they were not updated [14,69,71,72,73]. Therefore, we systematically reviewed all evidence on the role of any vitamin in the prevention and management of COVID-19 but also long-COVID, with the aim to provide healthcare professionals with a wider overview of the topic, including the latest available evidence, and considering simultaneously several aspects, such as different populations, settings, vitamin dosages, and route of administrations. We found that the prevention of COVID-19 was little investigated and in relation to vitamin D only. Moreover, given the contrasting findings, no clear conclusion could be drawn on the potential role of such vitamins in reducing the SARS-CoV-2 infection risk [25,26]. Similarly, even though vitamin D seems to provide some benefits in the long-term complications of COVID-19 [74], its effects in the prevention of long-COVID symptoms were poorly studied, together with the effects of vitamin co-administration in the management of COVID-19 patients, both of which did not show any robust result. However, considering the low quality of most trials included and the relatively safe profile of these substances, additional data could be produced in the near future, allowing a more conclusive judgment on the potential benefits of vitamin D as a prevention therapy and vitamin co-administration as a supplemental treatment for COVID-19 patients [75,76].

As for the efficacy of individual vitamin supplementation in managing SARS-CoV-2 infections, we found limited evidence on vitamins A and B, with most outcomes showing no additional benefit [29,30,31,32]. As for vitamin C, even though its role as an antioxidant agent [77,78] and necessary micronutrient for leukocyte function is well recognized [79], a few positive findings were reported in some trials but results were largely inconsistent in relation to most outcomes. Among these, all-cause mortality was the only one in which COVID-19 vitamin-supplemented individuals seemed to have significant benefits, in line with a recent meta-analysis that found reduced mortality rates after vitamin C administration in many diseases [80]. These results also align with other similar reviews conducted on COVID-19 subjects [13,15,71] and might be explained by the fact that patients with pneumonia, sepsis, and/or multiple organ failure, as happens in critically ill COVID-19 patients, have usually high oxidative stress [13,81]. Furthermore, vitamin C may have a role in various immunity and inflammation pathways against COVID-19 by regulating the growth and function of innate and adaptive immune cells, the production of antibodies, and the suppression of pro-inflammatory cytokines, potentially helping to balance the immune response and mitigate excessive inflammation [82,83]. Nevertheless, most randomized trials that investigated all-cause mortality incidence had some concerns or were at high risk of bias [37,39,41,42]. In addition, the treatment protocols were largely heterogeneous as for dose, route, and frequency of administration. For this reason, further studies are necessary to confirm our results, especially well-conducted RCTs that would contribute to populating the funnel plot and probably mitigate any potential presence of publication bias [84].

As for vitamin D, it is widely recognized for its role in the regulation of the immune system and how its deficiency is linked to inflammation. On the one hand, SARS-CoV-2 infection can cause an inflammation status leading to vitamin D deficiency [85], while, on the other hand, lower serum vitamin D levels may contribute to a dysregulation of the renin–angiotensin system and thus may increase the risk of developing a cytokine storm in COVID-19 [86,87]. For these reasons, it has been the object of many investigations in the last years [14,73], but its efficacy in COVID-19 management is still contradictory [68], as also found in this systematic review, in which the effects of vitamin D supplementation were mostly inconsistent. In this regard, it is worth mentioning the small sample sizes and low quality of the retrieved RCTs, together with the considerable heterogeneity among the included studies in terms of drug dosing, population characteristics, COVID-19 severity, and treatment strategies that may have affected these findings [68]. Interestingly, despite the hypothesis that individuals with low baseline 25-OH vitamin D levels may benefit to a different extent from the supplementation [68,88,89], we did not detect any increased effect, as already reported in the literature [68]. By contrast, this review found a reduced mortality rate in individuals supplemented regularly with vitamin D, a finding that aligns with a large population study in which an observed inverse association between habitual use of vitamin D supplements and COVID-19 infection was observed [90] and that suggests the presence of a positive effect of this vitamin on several inflammatory mechanisms induced by SARS-CoV-2 [90,91,92,93].

Additionally, it is known that treating SARS-CoV-2 infections is more effective in the early stages of the disease and therefore, by the time COVID-19 patients are hospitalized due to their critical conditions, the treatment is likely to be less effective [94]. This may be the case for vitamin administration as well, as both vitamin C and D help counterbalance the cytokine excess in the early phases of SARS-CoV-2 infection [83,87]. Moreover, the choice between different vitamin D forms may be relevant in this context due to their different pharmacokinetic profiles [95]. However, since most studies included patients already hospitalized, further research is needed to explore how the timing of vitamin supplementation affects the outcomes in COVID-19 patients.

However, as several trials are still ongoing or have not been published yet [96], new data could soon become available that may allow us to alleviate some uncertainty about the findings and may contribute to making the funnel plot more balanced. Alternatively, or in addition, given the high tolerability consistently mentioned across the studies, large RCTs with standardized recruitment and treatment protocols should be designed to further explore the clinical benefits of vitamin D supplementation in COVID-19 patients and better clarify the mechanisms by which it acts [68].

This study has some strengths and limitations. The main strength is the updated and comprehensive collection of data on the topic. Indeed, to the best of our knowledge, this is the first review that synthesizes available evidence on the role of any vitamin in both the prevention and management of COVID-19 and long-COVID, providing results for multivitamin complex and vitamins A, B, C, and D. Moreover, we studied all effects related to vitamin supplementation in patients with confirmed SARS-CoV-2 infection, including any immunological, hematological, laboratory, and clinical outcomes. By contrast, the limitations of the current review are mostly related to the primary studies included. Since most of them were conducted in low-middle income countries, enrolled a small sample size, and/or great proportions of individuals with vitamin deficiency, the generalizability of our findings may be limited. In addition, none of the studies provided information on the patients’ SARS-CoV-2 vaccination status or reported outcome data based on age groups and/or type or number of comorbidities, factors that could have been considered in the stratified analyses. Moreover, the included studies were conducted across different countries and at different times during the COVID-19 pandemic and they did not specify the SARS-CoV-2 variant responsible for the infections [97], nor did they assess the effect of supplements on the management or prevention of infections caused by different SARS-CoV-2 variants, limiting the opportunity to consider these aspects in the analyses. Furthermore, large heterogeneity in the recruitment and treatment protocols, as well as in the outcome definitions, was found, limiting the opportunity to provide a quantitative synthesis for most aspects. Lastly, the concerning quality of the trials, coupled with some uncertainties in the findings due to the potential presence of publication bias, made the interpretation of the results particularly challenging. This was not unexpected, given that, as highlighted by Honarmand et al. [98], several biases affected the largest part of the RTCs on COVID-19 management and prevention, making it imperative to prioritize rigorous trial design to address the demand for effective and safe treatment and prevention options during healthcare crises. For all these reasons, despite some positive findings, further and better-designed studies are needed to clarify the potential benefits of vitamin administration in relation to both the prevention and management of COVID-19 and long-COVID, using common pre-established daily dosages and standardized recruitment and intervention protocols.

## Figures and Tables

**Table 1:** Characteristics of the studies retrieved from the literature search and included in the systematic review that analyzed vitamin supplementation and prevention of COVID-19 and/or long-COVID.

Author, Year	Country	Population	Vitamin Status at Baseline	Vitamin Dose, Form, Route of Administration	Frequency of Administration	Follow-Up Time	Overall Risk of Bias
COVID-19							
Jolliffe, 2022 ^#^ [25]	United Kingdom	5979 non-hospitalized subjects, adults, 77% female	25-OH-vitamin D levels [mean ± SD)]: Group I: 40.9 ± 16.4 nmol/L Group II: 41.5 ± 18.0 nmol/L Group III: >75 nmol/L 100% vitamin D deficient subjects in Group I and Group II (≤75 nmol/L) 100% vitamin D non-deficient subjects in Group III (>75 nmol/L)	Vitamin D3 3200 or 800 IU, oral	Once daily for 6 months	6 months	High
Villasis-Keever, 2022 [26]	Mexico	321 healthcare workers, SARS-CoV-2 negative, adults, 70% female	25-OH-vitamin D levels [median (IQR)]: Group I: 18.3 (14.6–22.9) ng/mL Group II: 17.1 (13.6–21.3) ng/mL 67% vitamin D deficient subjects (<20 ng/mL)	Cholecalciferol 4000 IU, oral	Once daily for 30 days	45 days	High
Long-COVID							
Jolliffe, 2022 ^#^ [25]	United Kingdom	5979 non-hospitalized subjects, adults, 77% female	25-OH-vitamin D levels [mean ± SD)]: Group I: 40.9 ± 16.4 nmol/L Group II: 41.5 ± 18.0 nmol/L Group III: >75 nmol/L 100% vitamin D deficient subjects in Group I and Group II (≤75 nmol/L) 100% vitamin D non-deficient subjects in Group III (>75 nmol/L)	Vitamin D3 3200 or 800 IU, oral	Once daily for 6 months	6 months	High

COVID-19: Coronavirus disease 2019. IQR: interquartile range. SD: standard deviation. ^#^ This trial evaluated two different outcomes.

**Table 2:** Characteristics of the studies included in the systematic review that analyzed the effects of vitamin supplementation in the management of COVID-19 by vitamin type.

Author, Year	Country	Population	Vitamin Status at Baseline	Vitamin Dose, Form, and Route of Administration	Frequency of Administration	Follow-Up Time	Overall Risk of Bias
Vitamin co-administration							
Beigmohammadi, 2021 [9]	Iran	60 ICU-hospitalized patients with severe COVID-19, adults (20–60 years), 49% female	Group I: Vitamin A (median ± IQR): 0.20 ± 0.20 ng/mL Vitamin D (median ± IQR): 22.00 ± 9.07 ng/mL Vitamin E (mean ± SD): 11.30 ± 3.60 µg/mL Vitamin C (median ± IQR): 0.20 ± 0.20 mg/dL, Vitamin B9 (mean ± SD): 7.90 ± 3.80 ng/mL Vitamin B12 (mean ± SD): 480.34 ± 292.7 pg/mL Group II: Vitamin A (median ± IQR): 0.20 ± 0.22 ng/mL Vitamin D (median ± IQR): 22.00 ± 12.35 ng/mL Vitamin E (mean ± SD): 11.01 ± 2.53 µg/mL Vitamin C (median ± IQR): 0.10 ± 0.10 mg/dL Vitamin B9 (mean ± SD): 6.54 ± 3.10 ng/mL Vitamin B12 (mean ± SD): 521.25 ± 324.67 pg/mL	Vitamin A: 25,000 IU, intravenous Vitamin D: 600,000 IU, intravenous Vitamin E: 300 IU, intravenous Vitamin C: 0.5 g, intravenous Vitamin B-complex: thiamine nitrate 3.1 mg, sodium riboflavin phosphate 4.9 mg, nicotinamide 40 mg, pyridoxine hydrochloride 4.9 mg, sodium pantothenate 16.5 mg, biotin 60 μg, folic acid 400 μg, and cyanocobalamin 5 μg, intravenous	Vitamin A: once daily for 7 days Vitamin D: once Vitamin E: 2 times Vitamin C: 4 times/day for 7 days Vitamin B-complex: once daily for 7 days	7 days	SC
Hakamifard, 2022 [27]	Iran	73 hospitalized patients with non-severe COVID-19, adults (≥18 years), 37% female	NA	Vitamin C: 1 g, oral Vitamin E: 400 IU, oral	Once daily until discharge	Until hospital discharge	High
Leal-Martínez, 2022 [28]	Mexico	80 hospitalized patients with severe COVID-19, adults (30–75 years), 35% female	NA	Vitamin C: 2 g, oral Vitamin D: 4000 IU, oral Vitamin B-complex: thiamin 100 mg, cyanocobalamin 10 mg, pyridoxine 100 mg, folic acid 5 mg, intramuscular	Vitamin C and D: 2 times/day for 21 days Vitamin B-complex: once daily for 5 days	40 days	Low
Vitamin A							
Rohani, 2022 [29]	Iran	182 outpatients with COVID-19, adults (18–75 years), 41.8% female	NA	Vitamin A 25,000 IU, oral	Once daily for 10 days	Ten days	SC
Somi, 2022 [30]	Iran	30 hospitalized patients with COVID-19, adults (≥18 years), 36.7% female	NA	Vitamin A 50,000 IU, intramuscular	Once daily for two weeks	Until hospital discharge	Low
Vitamin B							
Majidi, 2022 [31]	Iran	85 ICU-hospitalized patients with severe COVID-19, adults (35–85 years), 49.0% female	NA	Vitamin B complex, including thiamine (10 mg), riboflavin (4 mg), nicotinamide (40 mg), and dexpanthenol (6 mg), intramuscular	Daily for two weeks	Two weeks	SC
Hu, 2022 [32]	China	24 hospitalized patients with COVID-19 and lymphopenia, adults (18–85 years), 54.2% female	NA	Nicotinamide 100 mg, not specified the route of administration	Five times daily for 2 days	Two days	High
Vitamin C							
Coppock, 2022 [33]	United States	66 hospitalized patients COVID-19, adults (≥18 years), 50% female	NA	Ascorbic acid 0.3 g/kg on day 0, 0.6 g/kg on day 1, 0.9 g/kg on days 2–5, intravenous	Once daily for 5 days	Until hospital discharge	Low
Fogleman, 2022 [34]	United States	104 non-hospitalized patients with mild or moderate COVID-19, adults (≥40 years), 80% female	NA	1 g, oral	Once daily for 14 days	30 days	High
Jamali Moghadam Siahkali, 2021 [35]	Iran	60 hospitalized patients with severe COVID-19, adults (>18 years), 50% female	NA	1.5 g, intravenous	4 times/day for 5 days	Until hospital discharge	High
Kumar, 2022 [36]	India	60 ICU-hospitalized patients with moderate or severe COVID-19, adults (>18 years), 22% female	NA	1 g, intravenous	3 times/day for 4 days	Until hospital discharge	Low
Kumari, 2020 [37]	Pakistan	150 hospitalized patients with severe COVID-19, adults (mean age: 52.5 years), 43% female	NA	50 mg/kg, intravenous	Once daily, not specified the intervention duration	Until hospital discharge	High
Labbani-Mothlag, 2022 [38]	Iran	90 hospitalized patients with moderate or severe COVID-19, adults (>18 years), 43% female	NA	12 g, intravenous	2 times/day for 4 days	Until hospital discharge	Low
Majidi, 2021 [39]	Iran	120 ICU-hospitalized patients with severe COVID-19 and enteral nutrition, adults (35–75 years), 50% female	NA	0.5 g, oral (enteral feeding)	Once daily for 14 days	Until hospital discharge	High
Ried, 2021 [40]	Australia, Turkey	237 hospitalized patients with COVID-19, adults (22–99 years), 50% female	NA	50 mg/kg or 100 mg/kg, intravenous	4 times/day for 8 days	45 days	High
Tehrani, 2021 [41]	Iran	54 hospitalized patients with COVID-19, adults (>18 years), 61% female	NA	2 g, intravenous	4 times/day for 5 days	Until hospital discharge	High
Thomas, 2021 [42]	United States	214 non-hospitalized patients with COVID-19, adults (≥18 years), 62% female	NA	8 g, oral	2 or 3 times/day for 10 days	28 days	SC
Zhang, 2021 [43]	China	56 ICU-hospitalized patients with severe COVID-19, adults (18–80 years), 44% female	NA	12 g, intravenous	2 times/day for 7 days	28 days	SC
Vitamin D							
Abroug, 2023 [44]	Tunisia	117 individuals in isolation centers with COVID-19, adults (≥18 years), 44% female	NA	Cholecalciferol 200,000 IU/1 mL, oral	Single administration	1 year after admission	SC
Bishop, 2022 [45]	United States	171 non-hospitalized patients with mild or moderate COVID-19, adults (18–71 years), 46% female	25-OH-vitamin D levels (mean ± SD): Group I: 37.7 ± 12.1 ng/mL Group II: 37.1 ± 15.6 ng/mL	Calcifediol 30 μg, oral	300 μg on days 1 to 3 and 60 μg on days 4 to 27	28 days	High
Bugarin, 2023 [46]	Croatia	152 ICU-hospitalized patients with COVID-19, adults (>18 years), 72% female	25-OH-vitamin D levels [median (IQR)]: Group I: 25.3 (17.9–36.9) nmol/L Group II: 27.3 (16.0–37.3) nmol/L	Cholecalciferol 10,000 IU, oral	Once daily for ICU stay or at least 14 days	Until hospital discharge	Some concerns
Bychinin, 2022 [47]	Russia	110 ICU-hospitalized patients with severe COVID-19, adults (≥18 years), 50% female	25-OH-vitamin D levels [median (IQR)]: Group I: 9.6 (5.6–21.0) ng/mL Group II: 11.0 (8.6–15.0) ng/mL 51% severe vitamin D deficient patients (<10 ng/mL)	Cholecalciferol 60,000 IU or 5000 IU, oral	High dose once/week followed by low dose once/day until discharge	Until hospital discharge	Low
Cannata-Andía, 2022 [48]	Spain, Argentina, Guatemala, Chile	548 hospitalized patients with moderate to severe COVID-19, adults (>18 years), 37% female	25-OH-vitamin D levels [median (IQR)]: Group I: 17.0 (11.8–22.0) ng/mL Group II: 16.1 (11.5–22.0) ng/mL	Cholecalciferol 100,000 IU, oral	Single administration	Until hospital discharge	SC
De Niet, 2022 [49]	Belgium	50 hospitalized patients with COVID-19 and vitamin D deficiency, adults (≥18 years), 40% female	25-OH-vitamin D levels (mean ± SD): Group I: 17.9 ± 10.2 ng/mL, Group II: 6.9 ± 9.5 ng/mL, 100% Vitamin D deficient patients (<20 ng/mL)	Vitamin D3 25,000 IU, oral	Once daily for 4 consecutive days, then once weekly until hospital discharge or for 36 days	9 weeks	SC
Elamir, 2022 [50]	United States	50 hospitalized patients with COVID-19, adults (≥18 years), 50% female	NA	Calcitriol 0.5 μg, oral	Once daily for 14 days or hospital discharge	Until hospital discharge	SC
Entrenas Castillo, 2020 [51]	Spain	76 hospitalized patients with severe COVID-19, adults (≥18 years), 61% female	NA	Calcifediol 0.532 mg or 0.266 mg, oral	3 times/week in the first week, followed by once weekly until discharge or ICU admission	Until hospital discharge	Low
Karonova, 2022 [52]	Russia	129 hospitalized patients with COVID-19, adults (18–75 years), 49% female	25-OH-vitamin D levels [median (IQR)]: Group I: 17.8 (11.7–25.4) ng/mL Group II: 15.4 (11.0–22.9) ng/mL 81% vitamin D deficient patients (<30 ng/mL)	Cholecalciferol 50,000 IU, oral	2 times, on day 1 and day 8	9 days	SC
Maghbooli, 2021 [53]	Iran	106 hospitalized patients with COVID-19 and vitamin D deficiency, adults (>18 years), 40% female	25-OH-vitamin D levels (mean ± SD): Group I: 19 ± 8 ng/mL Group II: 18 ± 8 ng/mL 100% Vitamin D deficient patients (<30 ng/mL)	Calcifediol 25 μg (3000–6000 IU), oral	Once daily for 60 days	Two months after hospital discharge	High
Mariani, 2022 [54]	Argentina	218 hospitalized patients with mild or moderate COVID-19 and at least one risk factor for disease progression, adults (≥18 years), 47% female	25-OH-vitamin D levels [median (IQR)]: Group I: 32.5 (27.2–44.2) ng/mL Group II: 30.5 (22.5–36.2) ng/mL	Cholecalciferol 500,000 IU, oral	Single administration	Until hospital discharge	High
Murai [§], 2021 A [55]	Brazil	240 hospitalized patients with moderate or severe COVID-19, adults (≥18 years), 43% female	25-OH-vitamin D levels (mean ± SD): Group I: 21.2 ± 10.1 ng/mL Group II: 20.6 ± 8 ng/mL 48% vitamin D deficient patients (<20 ng/mL)	Vitamin D3 200,000 IU, oral	Single administration	Until hospital discharge	Low
Murai [§], 2021 B [56]	Brazil	32 hospitalized patients with moderate or severe COVID-19 and severe vitamin D deficiency, adults (≥18 years), 44% female	25-OH-vitamin D levels (mean ± SD): Group I: 7.7 ± 1.6 ng/mL Group II: 7.7 ± 1.9 ng/mL 100% severe vitamin D deficient patients (<10 ng/mL)				High
Fernandes [§], 2022 [57]	Brazil	240 hospitalized patients with moderate or severe COVID-19, adults (≥18 years), 43% female	25-OH-vitamin D levels (mean ± SD): Group I: 21.1 ± 10.1 ng/mL Group II: 20.2 ± 8.1 ng/mL			4 months	High
Rastogi, 2021 [58]	India	40 hospitalized patients with mild or moderate COVID-19, adults, 50% female	25-OH-vitamin D levels [median (IQR)]: Group I: 8.6 (7.1–13.1) ng/mL Group II: 9.54 (8.1–12.5) ng/mL 100% severe vitamin D deficient patients (<20 ng/mL)	Cholecalciferol 60,000 IU, oral	Once daily for 7 days, followed by once weekly for the following 7 days (if 25-OH-vitamin D levels > 50 ng/mL) or once daily for the following 7 days (if 25-OH-vitamin D levels <50 ng/mL)	21 days	High
Sánchez-Zuno, 2021 [59]	Mexico	42 non-hospitalized patients with mild or moderate COVID-19, adults (>18 years), 52% female	25-OH-vitamin D levels [median (IQR)]: Group I: 20.2 (12.2–45.9) ng/mL Group II: 23.4 (12.1- 45.6) ng/mL 80% vitamin D deficient or insufficient patients (<30 ng/mL)	Vitamin D3 10,000 IU, oral	Once daily for 14 days	14 days	SC
Zurita-Cruz, 2022 [60]	Mexico	45 hospitalized patients with moderate COVID-19, pediatric patients (1–17 years), 60% female	25-OH-vitamin D levels [median (IQR)]: Group I: 13.8 (10.8–18.4) ng/mL Group II: 11.4 (8.7–13.1) ng/mL 100% severe Vitamin D deficient patients (<20 ng/mL)	Vitamin D3 1000 IU/day among children < 1 year or 2000 IU/day among children 1–17 years, oral	Once daily for 7–14 days	14 days	High

§ studies with the same symbol included participants from the same trial. COVID-19: coronavirus disease 2019. ICU: intensive care unit. IQR: interquartile range. IU: international unit. SC: some concerns. SD: standard deviation.

**Table 3:** Main effects of vitamin administration in the prevention of COVID-19 and/or long-COVID.

Author, Year	Intervention	Clinical Outcomes	Mortality	Side Effects or Adverse Events
COVID-19				
Jolliffe, 2022 [25] ^#^	Group I: High dose vitamin D Group II: Low dose vitamin D Group III: No vitamin	− Non-significant difference in the proportion of subjects developing SARS-CoV-2 infection, subjects hospitalized for COVID-19, subjects hospitalized for COVID-19 requiring respiratory support	NA	− Non-significant difference in the proportion of subjects developing serious effects
Villasis-Keever, 2022 [26]	Group I: vitamin D3 Group II: Placebo	− Significant lower proportion of subjects developing SARS-CoV-2 infection in Group I	NA	− Non-significant difference in the proportion of subjects developing side effects
Long-COVID				
Jolliffe, 2022 [25] ^#^	Group I: High dose vitamin D Group II: Low dose vitamin D Group III: No vitamin	− Non-significant difference in the proportion of subjects developing test-confirmed COVID-19 who reported symptoms lasting >4 weeks or on-going symptoms at month 6 − Non-significant differences in the MRC dyspnea score, FACIT Fatigue Scale score, Post-COVID Physical Health Symptom score among subjects developing test-confirmed COVID-19 who reported ongoing symptoms at month 6	− Non-significant differences in 6-month COVID-19 and all-cause mortality	− Non-significant difference in the proportion of subjects developing serious effects

COVID-19: Coronavirus disease 2019. FACIT: functional assessment of chronic illness therapy. MRC: United Kingdom Medical Research Council. NA: not assessed. ^#^ this trial evaluated two different outcomes.

**Table 4:** Main effects of supplementation with multivitamins and vitamin A, vitamin B, and vitamin C in the management of COVID-19.

Author, Year	Intervention	Immunological, Hematological, and Laboratory Outcomes	Clinical Outcomes and Mortality	Length of Hospitalization	Side Effects or Adverse Events
Vitamin co-administration					
Beigmohammadi, 2021 [9]	Group I: Vitamin A + Vitamin D + Vitamin E + Vitamin C + Vitamin B (in combination) Group II: No vitamin	− Non-significant differences in WBC count, neutrophil count, IFN-γ levels on day 7 − Significant lower ESR, CRP, IL-6, and TNF-α levels in Group I on day 7	− Significant lower SOFA score in Group I on day 7	− Significant lower proportion of patients with hospitalization > 7 days in Group I	− No side effect detected
Hakamifard, 2022 [27]	Group I: Vitamin C + Vitamin E + SoC Group II: SoC	− Non-significant difference in WBC and platelet count and LDH levels until discharge	− Non-significant difference in ICU-admission rate − Non-significant difference in temperature, pulse rate and SpO_2_ at any point until discharge − Significant lower respiratory rate in Group I on day 4 and 8	− Non-significant difference in mean length of hospitalization	NA
Leal-Martínez, 2022 [28]	Group I: Vitamin C + Vitamin D + Vitamin B (in combination) Group II: No vitamin	− Significant higher hydric balance in Group I on day 3 − Significant lower gastrointestinal distension in Group I on day 3	− Significant lower mortality in Group I − Significant higher saturation without supplementary oxygen in Group I on day 40 − Non-significant need for home oxygen, time of home oxygen, post-COVID syndrome, weight decrease, gastrointestinal symptoms on day 40	NA	NA
Vitamin A					
Rohani, 2022 [29]	Group I: Vitamin A + SoC Group II: SoC + placebo	− Significant lower WBC count and CRP levels in Group I	− Significant lower persistence of fever, body ache, weakness, fatigue in Group I	NA	NA
Somi, 2022 [30]	Group I: Vitamin A + SoC Group II: SoC	NA	− Non-significant difference in the proportion of patients admitted in ICU − Non-significant difference in the proportion of patients requiring respiratory support − Non-significant difference in treatment strategies − Non-significant difference in the time to clinical response − Non-significant difference in the need for IMV − Non-significant difference in mortality rate	− Non-significant difference in length of hospitalization	− No side effect detected
Vitamin B					
Majidi, 2022 [31]	Group I: Vitamin B complex Group II: nutritional support without Vitamin B complex	− Non-significant difference in BG, serum electrolytes, kidney function, BG − Non-significant difference in arterial blood gas parameters − Non-significant difference in blood clotting functions, CBC, and coagulation parameters	− Non-significant difference in MAP and O_2_ saturation	NA	NA
Hu, 2022 [32]	Group I: Vitamin B + SoC Group II: SoC	− Non-significant difference in the lymphocyte count − Non-significant difference in WBC and RBC count, hemoglobin, and PCR	− Non-significant difference in clinical aggravation − Non-significant difference in mortality rate	NA	NA
Vitamin C					
Coppock, 2022 [33]	Group I: Vitamin C + SoC Group II: SoC	NA	− Non-significant difference in the proportion of patients achieving clinical improvement within 72 h of randomization − Non-significant difference in the proportion of patients developing clinical decline within 36 h of randomization − Non-significant difference in the proportion of patients achieving a 50% supplemental oxygen reduction, a 50% bronchodilator use reduction − Non-significant difference in the proportion of patients being discharged within 72 h of randomization − Non-significant difference in the time to achieve a 50% supplemental oxygen reduction − Non-significant difference in the proportion of patients with any fever	− Non-significant difference in length of hospitalization	− Non-significant difference in the proportion of patients developing serious side effects
Fogleman, 2022 [34]	Group I: Vitamin C Group II: Melatonin Group III: Placebo	NA	− Non-significant difference in WURSS symptom severity score between Group I and Group III on days 1 to 3, days 3 to 9 and days 9 to 14 − Non-significant difference in WURSS quality-of-life score between Group I and Group III on days 3 to 9	NA	NA
Jamali Moghadam Siahkali, 2021 [35]	Group I: Vitamin C + SoC Group II: SoC	NA	− Non-significant difference in the proportion of patients requiring intubation and patients requiring corticosteroid therapy − Significant lower temperature and higher SpO_2_ levels in Group I on day 3 − Non-significant difference in temperature and SpO_2_ levels at discharge	− Overall patients: − Significant longer length of hospitalization in Group I − ICU-admitted patient subgroup: − Non-significant difference in ICU length of stay	− No side effect detected
Kumar, 2022 [36]	Group I: Vitamin C + SoC Group II: Placebo + SoC	NA	− Non-significant difference in SOFA score, MAP, and respiratory rate on day 3, 5, 7 and 9 − Non-significant difference in invasive mechanical ventilation, non-invasive mechanical ventilation, high flow nasal cannula time and vasopressor use	NA	NA
Kumari, 2020 [37]	Group I: Vitamin C + SoC Group II: SoC	NA	− Significant shorter time to resolution of COVID-19 symptoms in Group I − Non-significant difference in the proportion of patients requiring mechanical ventilation	− Significant shorter length of hospitalization in Group I	NA
Labbani-Mothlag, 2022 [38]	Group I: Vitamin C + SoC Group II: Placebo + SoC	− Non-significant difference in CRP, ferritin, NLR	− Non-significant difference in SF ratio, SOFA score, NEWS score, and Ordinal Scale for Clinical Improvement on day 3 and 5 − Non-significant difference in ICU admission rate	− Non-significant difference in length of hospitalization	NA
Majidi, 2021 [39]	Group I: Vitamin C Group II: No vitamin	− Non-significant difference in RBC, WBC, platelet count, ESR, hemoglobin levels, INR levels, arterial pH, bicarbonate and pCO_2_ levels, potassium, sodium, calcium, phosphorus, BUN, creatinine, blood glucose, and albumin levels	− Non-significant difference in SpO_2_ and MAP levels, GCS score	NA	− Non-significant difference in the proportion of patients developing side effects
Ried, 2021 [40]	Group I: Vitamin C + Vitamin D + SoC Group II: Vitamin D + SoC	NA	− Significant higher proportion of patients reaching total recovery on day 15 in Group I − Non-significant difference in the proportion of patients reaching total recovery on day 45	NA	− Non-significant difference in the proportion of patients developing side effects in the first 10 days from enrollment
Tehrani, 2021 [41]	Group I: Vitamin C + SoC Group II: SoC	− Non-significant difference in lymphocyte count and CRP levels on day 6	− Significant higher oxygen saturation on day 6 in Group I − Significant lower respiratory rate on day 6 in Group I − Significant lower proportion of patients with severe lung parenchymal involvement on day 6 in Group I	− Non-significant difference in length of hospitalization	NA
Thomas, 2021 [42]	Group I: Vitamin C Group II: Zinc gluconate Group III: Zinc + Vitamin C Group IV: SoC	NA	− Non-significant difference in time to reach a 50% reduction in symptom severity score, in time to reach a symptom severity score of 0, in cumulative severity score on day 5, in the proportion of hospitalized patients	NA	− Significant higher proportion of patients developing any side effect in Group I
Zhang, 2021 [43]	Group I: Vitamin C + SoC Group II: Placebo + SoC	− Non-significant difference in WBC, neutrophil, lymphocyte count, procalcitonin levels, CRP levels on day 7 − Significant lower IL-6 levels on day 7 in Group I	− Non-significant difference in the proportion of patients with condition deterioration or condition improvement on day 7 − Non-significant difference in SOFA score changes on day 7 − Non-significant difference in invasive mechanical ventilation, non-invasive mechanical ventilation, and high flow nasal cannula time − Significant higher P/F ratio change on day 7 in Group I − Non-significant difference in MAP change on day 7	− Non-significant difference in ICU length of stay and total length of hospitalization	− Non-significant difference in the proportion of patients developing infusion-related side effects

BG: blood glucose. BUN: blood urea nitrogen. CBC: cell blood count. CRP: C-reactive protein. CS: Glasgow Coma Scale. ESR: erythrocyte sedimentation rate. ICU: intensive care unit. IFN: interferon. IL: interleukin. IMV: invasive mechanical ventilation. INR: international normalized ratio of prothrombin time of blood coagulation. LDH: lactate dehydrogenase. MAP: mean arterial pressure. NA: not assessed. NEWS: national early warning score. NLR: neutrophil-to-lymphocyte ratio. RBC: red blood cell. SoC: standard of care. SOFA: sequential organ failure assessment. SF ratio: SpO_2_/FiO_2_ ratio. TNF: tumor necrosis factor. WBC: white blood cell. WURSS: Wisconsin Upper Respiratory Symptom Survey.

**Table 5:** Main effects of vitamin D administration in the management of COVID-19.

Author, Year	Intervention	Immunological, Hematological, and Laboratory Outcomes	Clinical Outcomes	Length of Hospitalization	Side Effects or Adverse Events
Abroug, 2023 [44]	Group I: Vitamin D Group II: Placebo	− Significant longer duration of viral RNA conversion in Group I	− Non-significant difference in the proportion of patients with persistent COVID-19 symptoms at 1 year − Non-significant difference in the proportion of patients developing a second SARS-CoV-2 infections at 1 year	NA	NA
Bishop, 2022 [45]	Group I: Vitamin D Group II: Placebo	− Non-significant difference in neutrophil and lymphocyte count on day 14, calcium and phosphorus levels on day 14 − Non-significant difference eGFR on day 14	− Non-significant difference in time to resolution of COVID-19 symptoms	NA	NA
Bugarin, 2023 [46]	Group I: Vitamin D Group II: SoC	− Non-significant difference in NLR, CRP, procalcitonin, fibrinogen, and D-dimer levels	− Non-significant difference in WHO progression scale on day 28 − Non-significant difference in number of days on respiratory support − Non-significant difference in occurrence of bacterial superinfections	− Non-significant difference in hospital and ICU length of stay	− No side effect detected
Bychinin, 2022 [47]	Group I: Vitamin D Group II: Placebo	− Significant higher NLR, NK and NKT cell count in Group I on day 7 − Non-significant difference in lymphocytes count, IL-6, PCR, and procalcitonin levels on day 7	− Non-significant difference in mechanical ventilation requirement, incidence of healthcare-associated infections, norepinephrine use − Significant higher proportion of positive blood cultures in Group I − Significant higher number of days undergoing mechanical ventilation	− Significant higher length of hospitalization in Group I	− No side effect detected
Cannata-Andía, 2022 [48]	Group I: Vitamin D Group II: No vitamin	− Non-significant difference in creatinine, CRP, albumin, hemoglobin, LDH, leucocytes, ferritin, calcium, and phosphate at discharge	− Non-significant difference in ICU admission rate − Non-significant difference in the proportion of patients with COVID-19 symptoms at discharge	− Non-significant difference in length of hospitalization	NA
De Niet, 2022 [49]	Group I: Vitamin D + SoC Group II: Placebo + SoC	NA	− Non-significant difference in the proportion of patients admitted to ICU, patients requiring supplemental oxygen or respiratory support − Non-significant difference in time until absence of fever or duration of respiratory support − Significant shorter duration of oxygen supplementation among patients requiring supplemental oxygen in Group I − Significant higher proportion of patients with clinical improvement in Group I on day 7	− Overall patients: − Significant shorter length of hospitalization in Group I − ICU-admitted patient subgroup: − Non-significant difference in ICU length of stay	− No side effect detected
Elamir, 2022 [50]	Group I: Vitamin D + SoC Group II: SoC	NA	− Non-significant difference in ICU admission rate, endotracheal intubation, readmission within 30 days − Significant greater change in SaO_2_/FiO_2_ ratio between discharge and admission in Group I	− Non-significant difference in length of hospitalization	− No side effect detected
Entrenas Castillo, 2020 [51]	Group I: Vitamin D + SoC Group II: SoC	− Non-significant difference in IL-6, D-Dimer, CRP and LDH levels, and lymphocyte count	− Significant lower ICU admission rate in Group I	NA	− Non-significant difference in the proportion of patients developing side effects
Karonova, 2022 [52]	Group I: Vitamin D Group II: No vitamin	− Vitamin D deficient/insufficient patient subgroup: − Significant higher neutrophil and lymphocyte counts, double-negative B cell count in Group I on day 9 − Significant lower CRP levels, transitional B cells mature, and naive B cells counts in Group I on day 9	− Vitamin D deficient/insufficient patient subgroup: − Non-significant difference in the proportion of patients admitted to ICU, requiring supplemental oxygen − Non-significant difference in SpO_2_ levels	− Vitamin D deficient/insufficient patient subgroup: − Non-significant difference in length of hospitalization	NA
Maghbooli, 2021 [53]	Group I: Vitamin D + SoC Group II: Placebo + SoC	− Significant lower WBC and neutrophil count in Group I at discharge − Significant higher lymphocyte count in Group I at discharge − Non-significant difference in platelet count at discharge − Non-significant difference in WBC, neutrophil, lymphocyte, and platelet count at month 1 and 2 after discharge − Non-significant difference in LDH levels at discharge	− Non-significant difference in the proportion of patients admitted to ICU, requiring supplemental oxygen, requiring mechanical ventilation, requiring antiviral therapy, requiring corticosteroid therapy	− Overall patients: − Non-significant difference in length of hospitalization − ICU-admitted patient subgroup: − Non-significant difference in ICU length of stay	− No side effect detected
Mariani, 2022 [54]	Group I: Vitamin D Group II: Placebo	NA	− Non-significant difference in rSOFA and qSOFA score change, SpO_2_ level change − Non-significant difference in the proportion of patients admitted to ICU, desaturating, requiring mechanical ventilation	− Overall patients: − Non-significant difference in length of hospitalization − ICU-admitted patient subgroup: − Non-significant difference in ICU length of stay	− Non-significant difference in the proportion of patients developing at least one serious side effect
Murai [§], 2021 A [55]	Group I: Vitamin D Group II: Placebo	− Non-significant difference in RBC, WBC and platelet count, hemoglobin, calcium, phosphorus, PTH, creatinine, CRP, D-dimer, urea, total-, LDL- and HDL-cholesterol, triglycerides levels, and ESR	− Overall patients: − Non-significant difference in the proportion of patients admitted to ICU or requiring mechanical ventilation − Non-significant difference in the duration of mechanical ventilation − vitamin D-deficient patient subgroup: − Non-significant difference in the proportion of patients admitted to ICU or requiring mechanical ventilation − Non-significant difference in the duration of mechanical ventilation	− Overall patients: − Non-significant difference in length of hospitalization − vitamin D deficient patient subgroup: − Non-significant difference in length of hospitalization	− Non-significant difference in the proportion of patients developing side effects
Murai [§], 2021 B [56]	Group I: Vitamin D Group II: Placebo	NA	− Non-significant difference in the proportion of patients admitted to ICU or requiring mechanical ventilation	− Vitamin D deficient patient subgroup: − Non-significant difference in length of hospitalization	NA
Fernandes [§], 2022 [57]	Group I: Vitamin D Group II: Placebo	− Non-significant difference in WBC count and CRP levels, IL-1β, IL-4, IL-6, IL-10, IL-12p70, IL-17A, IFN-γ, TNF-α, IL-8, IP-10, MIP-1β, MCP-1, GM-CSF, and VEGF levels	NA	NA	NA
Rastogi, 2021 [58]	Group I: Vitamin D + SoC Group II: Placebo + SoC	− Non-significant difference in D-dimer, fibrinogen, CRP, and procalcitonin level change	− Significant higher proportion of SARS-CoV-2 negative subjects in Group I on day 21	NA	NA
Sánchez-Zuno, 2021 [59]	Group I: Vitamin D Group II: SoC	− Non-significant difference in the proportion of anti-SARS-CoV-2 IgM and/or IgG positive patients on day 7	− Non-significant difference in the proportion of SARS-CoV-2 positive patients on day 7 and day 14 − Significantly lower proportion of patients with at least 3 symptoms on day 7 and day 14 in Group I − Non-significant difference in the proportion of patients requiring drug treatment	NA	NA
Zurita-Cruz, 2022 [60]	Group I: Vitamin D + SoC Group II: SoC	NA	− Significant lower proportion of patients requiring superior ventilation modality in Group I	NA	− Non-significant difference in the proportion of patients developing side effects

§ Studies with the same symbol included participants from the same trial. CRP: C-reactive protein. eGFR: estimated glomerular filtration rate. IFN: interferon. GM-CSF: granulocyte-macrophage colony-stimulating factor. IL: interleukin. ICU: intensive care unit. LDH: lactate dehydrogenase. MCP-1: monocyte chemoattractant protein-1. MIP-1β: macrophage inflammatory protein-1β. NA: not assessed. NLR: neutrophil-to-lymphocyte ratio. NK: natural killer. NKT: natural killer T. RBC: red blood cell. SoC: standard of care. SOFA: sequential organ failure assessment. qSOFA: quick SOFA. rSOFA: respiratory SOFA. ESR: erythrocyte sedimentation rate. TLC: total leucocyte count. TNF: tumor necrosis factor. VEGF: vascular endothelial growth factor. WBC: white blood cell.

## Data Availability

Data are contained within the article and Supplementary Materials.

## Supplementary Materials

The following supporting information can be downloaded at https://www.mdpi.com/article/10.3390/nu16091345/s1. Table S1: Search string used in the systematic review by database; Table S2: Quality assessment of the articles included in the systematic review by alphabetic order. Revised Cochrane risk-of-bias tool for randomized trials (RoB2) was used; Figure S1: Funnel plot of randomized controlled trials comparing all-cause mortality between patients receiving Vitamin C vs. placebo or standard of care; Figure S2: Funnel plot of randomized controlled trials comparing all-cause mortality between patients receiving Vitamin D vs. placebo or standard of care.
