# Supplementary material for: The Efficacy of Multivitamin, Vitamin A, Vitamin B, Vitamin C, and Vitamin D Supplements in the Prevention and Management of COVID-19 and Long-COVID: An Updated Systematic Review and Meta-Analysis of Randomized Clinical Trials
Source: Nutrients. 2024 Apr 29;16(9):1345. doi: 10.3390/nu16091345 (PMC11085542; doi:10.3390/nu16091345)

**Table S1.** Search string used in the systematic review by database.

| Database | Search string |
|---|---|
| PUBMED | #**1** "Severe Acute Respiratory Syndrome Coronavirus 2" OR "SARS Coronavirus" OR "SARS Coronavirus 2" OR "SARS-CoV-2" OR "SARS-COV-2" OR "SARS CoV 2" OR "SARS COV 2" OR "SARS-CoV2" OR "SARS CoV2" OR "SARS-CoV" OR "SARS-CoV" OR "Novel Coronavirus" OR "nCoV" OR "2019 Novel Coronavirus" OR "2019-nCoV" OR "Wuhan Coronavirus" OR "COVID-19 " OR "COVID 19" OR "COVID19" OR "Coronavirus Disease" OR "coronavirus disease" OR "Coronavirus Disease 2019" OR "coronavirus disease 2019" OR "Coronavirus Disease-19" OR "coronavirus disease-19" OR "Coronavirus Disease 19" OR "coronavirus disease 19" OR "Novel Coronavirus Disease" OR "novel coronavirus disease" OR "Wuhan Seafood Market Pneumonia" OR "Wuhan seafood market pneumonia" OR "Coronavirus Infection" OR "coronavirus infection" OR "Novel Coronavirus Infection" OR "novel coronavirus infection" OR "SARS Coronavirus 2 Infection" OR "SARS coronavirus 2 infection" OR "SARS-CoV-2 Infection" OR "SARS-CoV-2 infection" OR "SARS-COV-2 Infection" OR "SARS-COV-2 infection" OR "SARS CoV 2 Infection" OR "SARS CoV 2 infection" OR "SARS COV 2 Infection" OR "SARS COV 2 infection" OR "nCoV Infection" OR "nCoV infection" OR "COVID-19"[Mesh] OR "SARS-CoV-2"[Mesh]<br>**AND**<br>#**2** vitamin* OR provitamin* OR micronutrient* OR "vitamin A" OR "carotenoids" OR "beta carotene" OR "β carotene" OR "carotene" OR "retinol" OR "vitamin D" OR "vitamin D2" OR "vitamin D3" OR "ergocalciferol" OR "paricalcitol" OR "cholecalciferol" OR "calcitriol" OR "calcifediol" OR "vitamin E" OR "tocopherol" OR "alpha tocopherol" OR "beta tocopherol" OR "gamma tocopherol" OR "tocotrienol" OR "vitamin K" OR "vitamin K1" OR "phytonadione" OR "vitamin K2" OR "menaquinone" OR "vitamin K3" OR "menadione" OR"vitamin B" OR "B complex" OR "vitamin B1" OR "thiamine" OR "vitamin B2" OR "riboflavin" OR "vitamin G" OR"vitamin B3" OR "niacin" OR "niacinamide" OR "nicotinic acid" OR "vitamin PP" OR"vitamin B5" OR "pantothenic acid" OR "pantothenate" OR"vitamin B6" OR "pyridoxine" OR "pyridoxal" OR "pyridoxamine" OR "vitamin B7" OR "biotin" OR "vitamin H" OR "vitamin B9" OR "folate" OR "folic acid" OR "l-methylfolate" OR "vitamin M" OR "vitamin B12" OR "cyanocobalamin" OR "methylcobalamin" OR "hydroxocobalamin" OR"vitamin C" OR "ascorbic acid" OR "ascorbate" OR "choline" OR "carnitine" OR "L-carnitine" OR "levocarnitine" OR "Vitamins"[Mesh] OR "Provitamins"[Mesh] OR "Micronutrients"[Mesh] OR "Vitamin A"[Mesh] OR "Carotenoids"[Mesh] OR "Vitamin D"[Mesh] OR "Vitamin E"[Mesh] OR "Vitamin K"[Mesh] OR "Vitamin B Complex"[Mesh] OR "Thiamine"[Mesh] OR "Riboflavin"[Mesh] OR "Niacinamide"[Mesh] OR "Pantothenic Acid"[Mesh] OR "Vitamin B 6"[Mesh] OR "Biotin"[Mesh] OR "Folic Acid"[Mesh] OR "Vitamin B 12"[Mesh] OR "Ascorbic Acid"[Mesh] OR "Choline"[Mesh] OR "Carnitine"[Mesh] |
| SCOPUS | TITLE-ABS-KEY("Severe Acute Respiratory Syndrome Coronavirus 2" OR "SARS Coronavirus" OR "SARS Coronavirus 2" OR "SARS-CoV-2" OR "SARS-COV-2" OR "SARS CoV 2" OR "SARS COV 2" OR "SARS-CoV2" OR "SARS CoV2" OR "SARS-CoV" OR "SARS-CoV" OR "Novel Coronavirus" OR "nCoV" OR "2019 Novel Coronavirus" OR "2019-nCoV" OR "Wuhan Coronavirus" OR "COVID-19 " OR "COVID 19" OR "COVID19" OR "Coronavirus Disease" OR "coronavirus disease" OR "Coronavirus Disease 2019" OR "coronavirus disease 2019" OR "Coronavirus Disease-19" OR "coronavirus disease-19" OR "Coronavirus Disease 19" OR "coronavirus disease 19" OR "Novel Coronavirus Disease" OR "novel coronavirus disease" OR "Wuhan Seafood Market Pneumonia" OR "Wuhan seafood market pneumonia" OR "Coronavirus Infection" OR "coronavirus infection" OR "Novel Coronavirus Infection" OR "novel coronavirus infection" OR "SARS Coronavirus 2 Infection" OR "SARS coronavirus 2 infection" OR "SARS-CoV-2 Infection" OR "SARS-CoV-2 infection" OR "SARS-COV-2 Infection" OR "SARS-COV-2 infection" OR "SARS CoV 2 Infection" OR "SARS CoV 2 infection" OR "SARS COV 2 Infection" OR "SARS COV 2 infection" OR "nCoV Infection" OR "nCoV infection") **AND** TITLE-ABS-KEY(vitamin* OR provitamin* OR micronutrient* OR "vitamin A" OR "carotenoids" OR "beta carotene" OR "β carotene" OR "carotene" OR "retinol" OR "vitamin D" OR "vitamin D2" OR "vitamin D3" OR "ergocalciferol" OR "paricalcitol" OR "cholecalciferol" OR "calcitriol" OR "calcifediol" OR "vitamin E" OR "tocopherol" OR "alpha tocopherol" OR "beta tocopherol" OR "gamma tocopherol" OR "tocotrienol" OR "vitamin K" OR "vitamin K1" OR "phytonadione" OR "vitamin K2" OR "menaquinone" OR "vitamin K3" OR "menadione" OR"vitamin B" OR "B complex" OR "vitamin B1" OR "thiamine" OR "vitamin B2" OR "riboflavin" OR "vitamin G" OR"vitamin B3" OR "niacin" OR "niacinamide" OR "nicotinic acid" OR "vitamin PP" OR"vitamin B5" OR "pantothenic acid" OR "pantothenate" OR"vitamin B6" OR "pyridoxine" OR "pyridoxal" OR "pyridoxamine" OR "vitamin B7" OR "biotin" OR "vitamin H" OR "vitamin B9" OR "folate" OR "folic acid" OR "l-methylfolate" OR |

| | |
|---|---|
| | "vitamin M" OR "vitamin B12" OR "cyanocobalamin" OR "methylcobalamin" OR "hydroxocobalamin" OR"vitamin C" OR "ascorbic acid" OR "ascorbate" OR "choline" OR "carnitine" OR "L-carnitine" OR "levocarnitine") |
| WEB OF SCIENCE | #**1** TS=("Severe Acute Respiratory Syndrome Coronavirus 2" OR "SARS Coronavirus" OR "SARS Coronavirus 2" OR "SARS-CoV-2" OR "SARS-COV-2" OR "SARS CoV 2" OR "SARS COV 2" OR "SARS-CoV2" OR "SARS CoV2" OR "SARS-CoV" OR "SARS-CoV" OR "Novel Coronavirus" OR "nCoV" OR "2019 Novel Coronavirus" OR "2019-nCoV" OR "Wuhan Coronavirus" OR "COVID-19 " OR "COVID 19" OR "COVID19" OR "Coronavirus Disease" OR "coronavirus disease" OR "Coronavirus Disease 2019" OR "coronavirus disease 2019" OR "Coronavirus Disease-19" OR "coronavirus disease-19" OR "Coronavirus Disease 19" OR "coronavirus disease 19" OR "Novel Coronavirus Disease" OR "novel coronavirus disease" OR "Wuhan Seafood Market Pneumonia" OR "Wuhan seafood market pneumonia" OR "Coronavirus Infection" OR "coronavirus infection" OR "Novel Coronavirus Infection" OR "novel coronavirus infection" OR "SARS Coronavirus 2 Infection" OR "SARS coronavirus 2 infection" OR "SARS-CoV-2 Infection" OR "SARS-CoV-2 infection" OR "SARS-COV-2 Infection" OR "SARS-COV-2 infection" OR "SARS CoV 2 Infection" OR "SARS CoV 2 infection" OR "SARS COV 2 Infection" OR "SARS COV 2 infection" OR "nCoV Infection" OR "nCoV infection")<br>**AND**<br>#**2** TS=(vitamin* OR provitamin* OR micronutrient* OR "vitamin A" OR "carotenoids" OR "beta carotene" OR "β carotene" OR "carotene" OR "retinol" OR "vitamin D" OR "vitamin D2" OR "vitamin D3" OR "ergocalciferol" OR "paricalcitol" OR "cholecalciferol" OR "calcitriol" OR "calcifediol" OR "vitamin E" OR "tocopherol" OR "alpha tocopherol" OR "beta tocopherol" OR "gamma tocopherol" OR "tocotrienol" OR "vitamin K" OR "vitamin K1" OR "phytonadione" OR "vitamin K2" OR "menaquinone" OR "vitamin K3" OR "menadione" OR"vitamin B" OR "B complex" OR "vitamin B1" OR "thiamine" OR "vitamin B2" OR "riboflavin" OR "vitamin G" OR"vitamin B3" OR "niacin" OR "niacinamide" OR "nicotinic acid" OR "vitamin PP" OR"vitamin B5" OR "pantothenic acid" OR "pantothenate" OR"vitamin B6" OR "pyridoxine" OR "pyridoxal" OR "pyridoxamine" OR "vitamin B7" OR "biotin" OR "vitamin H" OR "vitamin B9" OR "folate" OR "folic acid" OR "l-methylfolate" OR "vitamin M" OR "vitamin B12" OR "cyanocobalamin" OR "methylcobalamin" OR "hydroxocobalamin" OR"vitamin C" OR "ascorbic acid" OR "ascorbate" OR "choline" OR "carnitine" OR "L-carnitine" OR "levocarnitine") |
| MEDRXIV | **topic1** <- c("Severe Acute Respiratory Syndrome Coronavirus 2","SARS Coronavirus","SARS Coronavirus 2","SARS-CoV-2","SARS-COV-2","SARS CoV 2","SARS COV 2","SARS-CoV2","SARS CoV2","SARS-CoV","SARS-CoV","Novel Coronavirus","nCoV","2019 Novel Coronavirus","2019-nCoV","Wuhan Coronavirus","COVID-19 ","COVID 19","COVID19","Coronavirus Disease","coronavirus disease","Coronavirus Disease 2019","coronavirus disease 2019","Coronavirus Disease-19","coronavirus disease-19","Coronavirus Disease 19","coronavirus disease 19","Novel Coronavirus Disease","novel coronavirus disease","Wuhan Seafood Market Pneumonia","Wuhan seafood market pneumonia","Coronavirus Infection","coronavirus infection","Novel Coronavirus Infection","novel coronavirus infection","SARS Coronavirus 2 Infection","SARS coronavirus 2 infection","SARS-CoV-2 Infection","SARS-CoV-2 infection","SARS-COV-2 Infection","SARS-COV-2 infection","SARS CoV 2 Infection","SARS CoV 2 infection","SARS COV 2 Infection","SARS COV 2 infection","nCoV Infection","nCoV infection")<br>**topic2** <- c("vitamin*","provitamin*","micronutrient*","vitamin A","carotenoids","beta carotene","β carotene","carotene","retinol","vitamin D","vitamin D2","vitamin D3","ergocalciferol","paricalcitol","cholecalciferol","calcitriol","calcifediol","vitamin E","tocopherol","alpha tocopherol","beta tocopherol","gamma tocopherol","tocotrienol","vitamin K","vitamin K1","phytonadione","vitamin K2","menaquinone","vitamin K3","menadione","vitamin B","B complex","vitamin B1","thiamine","vitamin B2","riboflavin","vitamin G","vitamin B3","niacin","niacinamide","nicotinic acid","vitamin PP","vitamin B5","pantothenic acid","pantothenate","vitamin B6","pyridoxine","pyridoxal","pyridoxamine","vitamin B7","biotin","vitamin H","vitamin B9","folate","folic acid","l-methylfolate","vitamin M","vitamin B12","cyanocobalamin","methylcobalamin","hydroxocobalamin","vitamin C","ascorbic acid","ascorbate","choline","carnitine","L-carnitine","levocarnitine")<br>**query** <- list(topic1, topic2) |

| | |
|---|---|
| BIORXIV | **topic1** <- c("Severe Acute Respiratory Syndrome Coronavirus 2","SARS Coronavirus","SARS Coronavirus 2","SARS-CoV-2","SARS-COV-2","SARS CoV 2","SARS COV 2","SARS-CoV2","SARS CoV2","SARS-CoV","SARS-CoV","Novel Coronavirus","nCoV","2019 Novel Coronavirus","2019-nCoV","Wuhan Coronavirus","COVID-19 ","COVID 19","COVID19","Coronavirus Disease","coronavirus disease","Coronavirus Disease 2019","coronavirus disease 2019","Coronavirus Disease-19","coronavirus disease-19","Coronavirus Disease 19","coronavirus disease 19","Novel Coronavirus Disease","novel coronavirus disease","Wuhan Seafood Market Pneumonia","Wuhan seafood market pneumonia","Coronavirus Infection","coronavirus infection","Novel Coronavirus Infection","novel coronavirus infection","SARS Coronavirus 2 Infection","SARS coronavirus 2 infection","SARS-CoV-2 Infection","SARS-CoV-2 infection","SARS-COV-2 Infection","SARS-COV-2 infection","SARS CoV 2 Infection","SARS CoV 2 infection","SARS COV 2 Infection","SARS COV 2 infection","nCoV Infection","nCoV infection")<br>**topic2** <- c("vitamin*","provitamin*","micronutrient*","vitamin A","carotenoids","beta carotene","β carotene","carotene","retinol","vitamin D","vitamin D2","vitamin D3","ergocalciferol","paricalcitol","cholecalciferol","calcitriol","calcifediol","vitamin E","tocopherol","alpha tocopherol","beta tocopherol","gamma tocopherol","tocotrienol","vitamin K","vitamin K1","phytonadione","vitamin K2","menaquinone","vitamin K3","menadione","vitamin B","B complex","vitamin B1","thiamine","vitamin B2","riboflavin","vitamin G","vitamin B3","niacin","niacinamide","nicotinic acid","vitamin PP","vitamin B5","pantothenic acid","pantothenate","vitamin B6","pyridoxine","pyridoxal","pyridoxamine","vitamin B7","biotin","vitamin H","vitamin B9","folate","folic acid","l-methylfolate","vitamin M","vitamin B12","cyanocobalamin","methylcobalamin","hydroxocobalamin","vitamin C","ascorbic acid","ascorbate","choline","carnitine","L-carnitine","levocarnitine")<br>**query** <- list(topic1, topic2) |

**Table S2.** Quality assessment of the articles included in the systematic review by alphabetic order. Revised Cochrane risk-of-bias tool for randomized trials (RoB2) was used.

| Author, year | DOMAIN 1<br>Risk of bias arising from the randomization process | DOMAIN 2<br>Risk of bias due to deviations from the intended interventions (effect of assignment to intervention) | Risk of bias due to deviations from the intended interventions (effect of adhering to intervention) | DOMAIN 3<br>Missing outcome data | DOMAIN 4<br>Risk of bias in measurement of the outcome | DOMAIN 5<br>Risk of bias in selection of the reported result | Overall risk of bias |
|---|---|---|---|---|---|---|---|
| Abroug, 2023 | Low | Low | Some concerns | Low | Low | Low | Some concerns |
| Beigmohammadi, 2021 | Low | Low | Low | Some concerns | Low | Low | Some concerns |
| Bishop, 2022 | Low | Low | Some concerns | Low | Low | High | High |
| Bugarin, 2023 | Some concerns | Some concerns | Low | Low | Low | Low | Some concerns |
| Bychinin, 2022 | Low | Low | Low | Low | Low | Low | Low |
| Cannata-Andía, 2022 | Low | Low | Some concerns | Low | Low | Low | Some concerns |
| Coppock, 2022 | Low | Low | Low | Low | Low | Low | Low |
| De Niet, 2022 | Low | Low | Low | Some concerns | Low | Low | Some concerns |
| Entrenas Castillo, 2020 | Low | Low | Low | Low | Low | Low | Low |
| Elamir, 2022 | Low | Low | Some concerns | Low | Low | Low | Some concerns |
| Fernandes, 2022 | Some concerns | Low | Low | High | High | Some concerns | High |
| Fogleman, 2022 | Low | Some concerns | Some concerns | Some concerns | Some concerns | Some concerns | High |
| Hakamifard, 2022 | Some concerns | High | High | Some concerns | High | High | High |
| Hu, 2022 | Low | Low | Some concerns | Low | Some concerns | Some concerns | High |
| Jamali Moghadam Siahkali, 2021 | Some concerns | Some concerns | Some concerns | Some concerns | High | High | High |
| Jolliffe, 2022 | High | Low | Some concerns | Low | Low | Low | High |
| Karonova, 2022 | Some concerns | Low | Low | Low | Low | Some concerns | Some concerns |
| Kumar, 2022 | Low | Low | Low | Low | Low | Low | Low |
| Kumari, 2020 | High | High | High | High | High | High | High |
| Labbani-Mothlag, 2022 | Low | Low | Low | Low | Low | Low | Low |
| Leal-Martínez, 2022 | Low | Low | Low | Low | Low | Low | Low |
| Maghbooli, 2021 | High | Some concerns | High | Some concerns | Some concerns | High | High |
| Majidi, 2021 | Some concerns | Some concerns | Some concerns | Some concerns | Some concerns | Some concerns | High |
| Majidi, 2022 | Low | Low | Some concerns | Low | Low | Some concerns | Some concerns |
| Mariani, 2022 | Low | Some concerns | Some concerns | Some concerns | Some concerns | Some concerns | High |
| Murai, 2021 A | Low | Low | Low | Low | Low | Low | Low |

| | | | | | | | |
|---|---|---|---|---|---|---|---|
| Murai, 2021 B | Some concerns | Some concerns | Some concerns | Some concerns | Low | Low | High |
| Rastogi, 2021 | High | Low | Low | Low | Low | Some concerns | High |
| Ried, 2021 | High | Some concerns | Low | Low | High | High | High |
| Rohani, 2022 | Low | Low | Low | Low | Some concerns | Some concerns | Some concerns |
| Sánchez-Zuno, 2021 | Low | Low | NA | Low | Low | Low | Some concerns |
| Somi, 2022 | Low | Low | Low | Low | Low | Low | Low |
| Tehrani, 2021 | High | Some concerns | Low | Low | High | High | High |
| Thomas, 2021 | Some concerns | Low | Some concerns | Low | Low | Low | Some concerns |
| Villasis-Keever, 2022 | Some concerns | Low | Some concerns | Low | Low | Some concerns | High |
| Zhang, 2021 | Low | Low | Low | Low | Low | Some concerns | Some concerns |
| Zurita-Cruz, 2022 | High | High | Some concerns | Low | Low | High | High |

**Figure S1.** Funnel plot of randomized controlled trials comparing all-cause mortality between patients receiving Vitamin C vs. placebo or standard of care

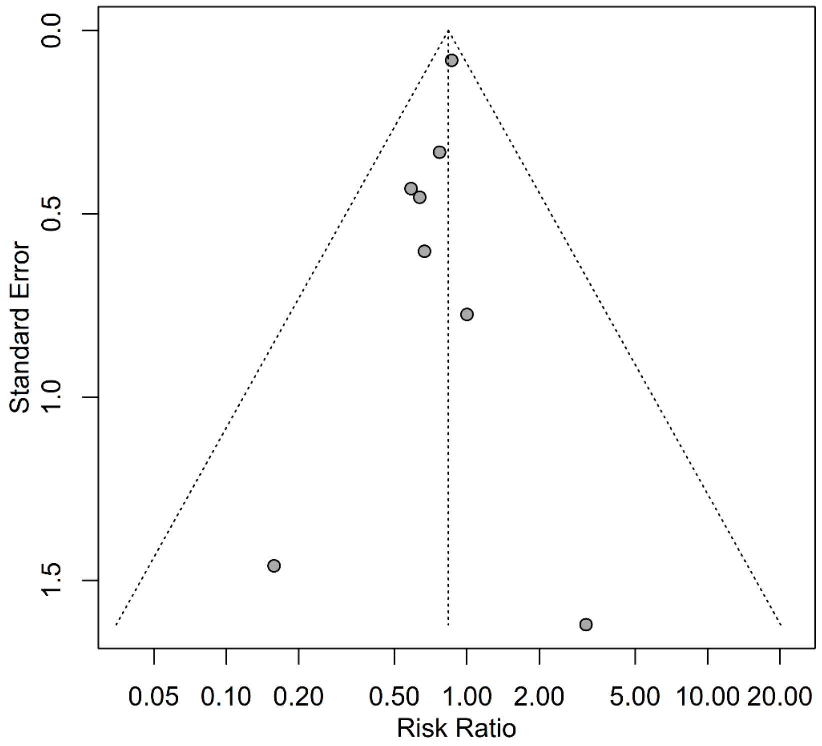

**Figure S2** Funnel plot of randomized controlled trials comparing all-cause mortality between patients receiving Vitamin D vs. placebo or standard of care.

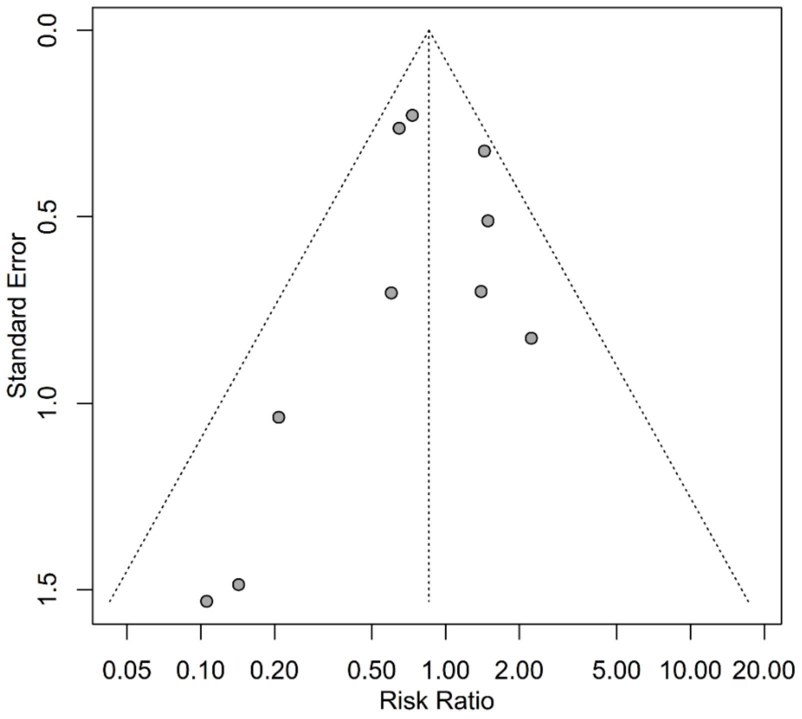

Supplement: Supplementary file 1 [file nutrients-16-01345-s001.zip › nutrients-2908560-supplementary.pdf]
